# EpiGraphDB: a database and data mining platform for health data science

**DOI:** 10.1093/bioinformatics/btaa961

**Published:** 2020-11-24

**Authors:** Yi Liu, Benjamin Elsworth, Pau Erola, Valeriia Haberland, Gibran Hemani, Matt Lyon, Jie Zheng, Oliver Lloyd, Marina Vabistsevits, Tom R Gaunt

**Affiliations:** MRC Integrative Epidemiology Unit, Bristol Medical School, University of Bristol, Bristol, UK; MRC Integrative Epidemiology Unit, Bristol Medical School, University of Bristol, Bristol, UK; MRC Integrative Epidemiology Unit, Bristol Medical School, University of Bristol, Bristol, UK; Cancer Genetics, Norwich Medical School, University of East Anglia, Norwich, UK; MRC Integrative Epidemiology Unit, Bristol Medical School, University of Bristol, Bristol, UK; MRC Integrative Epidemiology Unit, Bristol Medical School, University of Bristol, Bristol, UK; NIHR Bristol Biomedical Research Centre, University of Bristol, Bristol, UK; MRC Integrative Epidemiology Unit, Bristol Medical School, University of Bristol, Bristol, UK; MRC Integrative Epidemiology Unit, Bristol Medical School, University of Bristol, Bristol, UK; MRC Integrative Epidemiology Unit, Bristol Medical School, University of Bristol, Bristol, UK; MRC Integrative Epidemiology Unit, Bristol Medical School, University of Bristol, Bristol, UK; NIHR Bristol Biomedical Research Centre, University of Bristol, Bristol, UK

## Abstract

**Motivation:**

The wealth of data resources on human phenotypes, risk factors, molecular traits and therapeutic interventions presents new opportunities for population health sciences. These opportunities are paralleled by a growing need for data integration, curation and mining to increase research efficiency, reduce mis-inference and ensure reproducible research.

**Results:**

We developed EpiGraphDB (https://epigraphdb.org/), a graph database containing an array of different biomedical and epidemiological relationships and an analytical platform to support their use in human population health data science. In addition, we present three case studies that illustrate the value of this platform. The first uses EpiGraphDB to evaluate potential pleiotropic relationships, addressing mis-inference in systematic causal analysis. In the second case study, we illustrate how protein–protein interaction data offer opportunities to identify new drug targets. The final case study integrates causal inference using Mendelian randomization with relationships mined from the biomedical literature to ‘triangulate’ evidence from different sources.

**Availability and implementation:**

The EpiGraphDB platform is openly available at https://epigraphdb.org. Code for replicating case study results is available at https://github.com/MRCIEU/epigraphdb as Jupyter notebooks using the API, and https://mrcieu.github.io/epigraphdb-r using the R package.

**Supplementary information:**

Supplementary data are available at *Bioinformatics* online.

## 1 Introduction

The wealth and diversity of biomedical and population data now available to epidemiologists is enabling new discoveries and methods development in population health data science. However, harmonization and integration of data presents a challenge to researchers aiming to ‘triangulate’ evidence from different sources or uncover potential mechanistic pathways of disease development. This challenge can be tackled through the development of dedicated data integration platforms, which curate and combine data sources to enable integrative analyses, removing this burden from individual researchers.

One area in which data integration offers potential value is causal inference. Over the last two decades, Mendelian randomization (MR) (Davey Smith and Ebrahim, 2003) has risen to prominence as a key causal inference method. MR exploits genetic variants as causal ‘anchors’ (randomly allocated and invariant from conception) to estimate causal effects between an ‘exposure’ (risk factor) influenced by the genetic variant(s) and a health outcome. The approach has various assumptions, of which a key constraint is that the genetic variants should not pleiotropically affect the health outcome through a pathway other than the risk factor in question. The two-sample MR approach enables MR to be performed in situations where a risk factor (exposure) and an outcome are analysed for genetic association in separate studies (Pierce and Burgess, 2013), enabling the thousands of published genome-wide association study (GWAS) datasets (MacArthur *et al.*, 2017) to be leveraged for causal inference.

Database resources, such as the IEU OpenGWAS database (https://gwas.mrcieu.ac.uk) (Elsworth *et al.*, 2020a), and the linked MR-Base analytical platform (Hemani *et al.*, 2018) now enable systematic MR application using the MR Mixture of Experts approach (Hemani *et al.*, 2017). This ‘systems’ approach offers the capacity to standardize the evaluation of potential intervention targets, as we have recently demonstrated with the plasma proteome (Zheng *et al.*, 2020). However, such high-throughput approaches raise new challenges in the interpretation of the wealth of causal estimates generated. The integration of causal estimates with data from other sources is one way to tackle such challenges. Combining evidence with different biases (such as MR estimates, observational correlations and literature-mined experimental results) can provide more robust causal interpretation in an approach described as ‘triangulation’ (Lawlor *et al.*, 2017). Agreement between sources strengthens the case for causality, whilst disagreement helps identify sources of bias.

Integration of data also offers the scope to gain more mechanistic insight into complex networks of association. For example, linking phenotypic data with genetic variants and molecular pathway data may make it easier to identify potential intervention targets once a causal relationship has been established. Similarly, an extensive network of associations provides the opportunity to identify drug repositioning opportunities and on-target side effects for pharmaceutical targets.

Here, we describe EpiGraphDB (https://epigraphdb.org/), a database and analytical platform, that integrates trait relationships (causal, observational or genetic), literature-mined relationships, biological pathways, protein–protein interactions (PPIs), drug–target relationships and other data sources to support data mining of risk factor/disease relationships. In the following sections, we describe the EpiGraphDB platform and its biomedical and epidemiological resources, and then illustrate some potential applications of this platform through specific case studies.

## 2 Materials and methods

### 2.1The EpiGraphDB platform

**Fig. 1.**
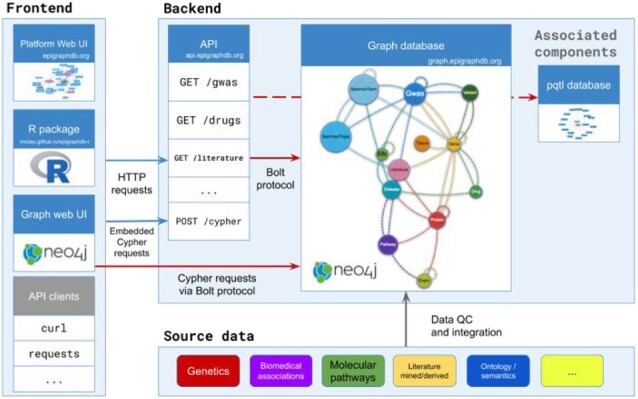
Architecture of the EpiGraphDB platform. Source datasets are integrated into a Neo4j graph database. Standard HTTP queries are processed through a RESTful API service, which can be called from any REST API client, including our R package epigraphdb. The web UI showcases main topics of the epidemiological evidence in EpiGraphDB and demonstrates the example API queries to get the underlying data

The EpiGraphDB platform (Fig. 1) integrates data from a range of biomedical and epidemiological sources into a Neo4j graph database and supports interactive and programmatic access using a variety of methods aimed at different needs. We provide a web user interface (UI) as a user-friendly entry point to the rich integrated resources that the platform offers, and users are able to programmatically query data via the application programming interface (API) web service or the client package in R. Finally, the Neo4j graph database can be directly queried in Cypher, which is supported as part of the API.

#### 2.1.1 Interactive access

As an entry point to the platform, the Web UI serves two primary purposes: (i) it showcases a selection of exemplar topics for the integrated biomedical evidence that EpiGraphDB provides and (ii) as an interactive interface it helps users in understanding how the queries of their requested data are structured, in order to assist their further use of EpiGraphDB. For example, the confounder topic view (https://epigraphdb.org/confounder) demonstrates the use of EpiGraphDB in investigating the potential confounders, mediators and colliders between exposures and outcomes. Aside from viewing the returned data in tabular format and its visualization in network diagrams, from the ‘Query’ tab users are able to see the underlying API call (using cURL, Python Requests and the epigraphdb R package) and Neo4j Cypher query to assist their further use of EpiGraphDB. In addition, users can use the Explore views (https://epigraphdb.org/explore) to browse and search EpiGraphDB and visit the Gallery (https://epigraphdb.org/gallery) for exemplar use cases.

#### 2.1.2 Programmatic access

EpiGraphDB can be queried via the API web service (https://api.epigraphdb.org), which includes a variety of accessible topic endpoints (as showcased on the Web UI) and other functionalities that enable further customized usage. In addition, we developed an R package epigraphdb (https://mrcieu.github.io/epigraphdb-r) to provide further ease of use for users to incorporate EpiGraphDB directly into their analytical pipelines in R, without having to be proficient in handling web requests or parsing response data. We discuss simple examples on how to query EpiGraphDB via the API or the R package in Supplementary Appendix S4, and further details on accessing EpiGraphDB are available in the platform documentation (https://docs.epigraphdb.org). In addition, we provide companion guides on replicating results of the Section 3 case studies in Jupyter notebooks and R package vignettes as well as a ‘getting started’ guide (the ‘getting started’ guide using Jupyter notebook can be found at https://github.com/MRCIEU/epigraphdb/blob/master/general-examples/getting-started-with-epigraphdb.ipynb. The guide using the R package can be found at https://mrcieu.github.io/epigraphdb-r/articles/getting-started-with-epigraphdb-r.html) on accessing EpiGraphDB data programmatically.

#### 2.1.3 Graph database and graph-based queries

At its core, EpiGraphDB stores integrated data using the Neo4j graph database. The graph database paradigm supports interpretable representation of biomedical information by storing data as relationships (e.g. associations, causal estimates and mappings) between entities (e.g. genes, proteins, diseases and genetic variants). Compared to a relational database architecture using structured query language, the use of Neo4j and the associated Cypher query language enables more natural representation of hypotheses as queries. For example, a hypothetical query for the causal effect for a risk factor on disease could be conceptualized in Cypher as a directed acyclic graph (r: RiskFactor)-[c: CausalEffect]->(d: Disease), which as is an exact representation of the epidemiological modelling. Although users are not required to know Cypher in order to use EpiGraphDB, the underlying Cypher query that is used to return user’s requested data via the Web UI, API or R package is returned as part of the response data, which can be further used as a baseline query for users to customize for their specific needs. Discussions on accessing EpiGraphDB in Cypher using the API and R package are available in Supplementary Appendix S4.

### 2.2Integration of epidemiological evidence

EpiGraphDB contains data from a range of biomedical and epidemiological sources (see criteria in Supplementary Appendix S2), with these data represented as nodes and relationships (we refer to a type of biomedical entity as a *meta node* (e.g. *(Gwas)* in Cypher notation) and a type of association as a *meta relationship* (e.g. *[MR]*), whereas a specific entity is referred to as a *node* (e.g. *(Gwas* *{id: ‘ieu-a-2’, trait: ‘Body mass index’})*) and a specific association as a *relationship* (e.g. [*Gwas* (*trait: ‘Body mass index’*)]*-*[*MR* {*beta, se, pval}*]*->(Gwas* {*trait: ‘Coronary heart disease’}*))) in a graph database. The relationships broadly represent: epidemiological relationships (e.g. genetic correlations) between phenotypes, biomedical mappings (e.g. genes to protein and pathways) and relationships derived from the biomedical literature. Table 1 reports a summary of the evidence available in EpiGraphDB.

**Table 1.** Summary of epidemiological evidence in EpiGraphDB

Category	Description		Sources	
Causal relationships	Pairwise MR between traits		MR-EvE (Hemani *et al.*, 2017)	
	pQTL/eQTL MR		xQTL (Zheng *et al.*, 2019)	
Association relationships	Genetic correlations		Neale Lab (Abbot *et al.*, 2020)	
	Observational correlations		EpiGraphDB inhouse^a^	
	GWAS top hits		OpenGWAS (Elsworth *et al.*, 2020a)	
	Polygenic risk score associations		PRS atlas (Richardson *et al.*, 2019)	
	PPIs		IntAct (Orchard *et al.*, 2014), STRING (Szklarczyk *et al.*, 2019)	
	Drug targets		Open targets (Carvalho-Silva *et al.*, 2019), CPIC (Relling and Klein, 2011), Druggable genome (Finan *et al.*, 2017)	
Molecular pathways	Pathway ontologies and molecular events		Reactome (Jassal *et al.*, 2019)	
	Gene expression for tissues		GTEx (The GTEx Consortium *et al.*, 2015)	
Literature-mined/derived evidence	Literature evidence of biomedical entities and mechanisms		SemMedDB (Kilicoglu *et al.*, 2012), MELODI (Elsworth *et al.*, 2018), MetaMap (Demner-Fushman *et al.*, 2017), Monarch (Mungall *et al.*, 2017)	
	Mapping of biomedical entities to literature terms			
Ontology and semantic relationships	Mapping of biomedical entities to ontology terms		EFO (Malone *et al.*, 2010), SemMedDB (Kilicoglu *et al.*, 2012), Vectology (Elsworth *et al.*, 2020b), MELODI (Elsworth *et al.*, 2018),	
	Semantic similarities of biomedical entities		Vectology (Elsworth *et al.*, 2020b)	
Entity metrics^b^	Meta nodes	Meta relationships	Nodes	Relationships
	14	42	32 969 103	84 181 124

*Note*: Detailed discussion on data integration and how these biomedical entities and associations are represented in EpiGraphDB are available in the Supplementary Appendices S1 and S2.

a Further details on the inhouse results by EpiGraphDB members are available from Supplementary Appendix S2.

b Information and metrics are based on latest version of EpiGraphDB platform (version 0.3.0, April 21, 2020).

We combined data from over 20 independent sources. All datasets required some level of processing in preparation for loading into EpiGraphDB. Detailed information including sources of data, processing steps and data ingest method are described in the Supplementary Appendix S2 and further in the platform documentation (https://docs.epigraphdb.org).

## 3 Results

The data integrated within EpiGraphDB offer a wide array of potential opportunities for data mining and analysis. Here, we present three case studies which illustrate some of the potential that is afforded by EpiGraphDB for new knowledge discovery. These do not, however, represent the full extent of the data or potential of the platform, which is provided as an open resource for the reader to use for their own novel research investigations.

In case study 1, we explore the potential of pathway data to characterize pleiotropy of genetic instruments used to generate causal estimates of the effect of protein levels on disease outcomes. Case study 2 seeks to identify alternative drug targets using PPI data in conjunction with causal estimates of protein levels on disease outcomes as well as literature-mined/derived evidence. Case study 3 uses knowledge extracted from the scientific literature to identify potential mechanistic pathways linking causal risk factors to diseases. We discuss the general steps to replicate these case studies in Supplementary Appendix S5 and users are encouraged to use the Jupyter notebooks and R package to replicate and modify the analyses.

### 3.1Distinguishing vertical and horizontal pleiotropy for SNP–protein associations

**Fig. 2.**
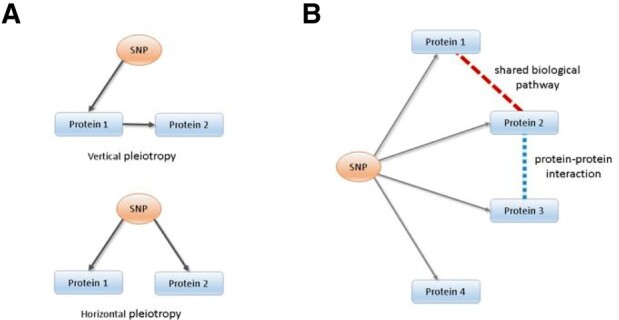
Distinguishing vertical and horizontal pleiotropy using EpiGraphDB. (**A**) Concept of vertical and horizontal pleiotropy using SNP–proteins relationship as an example. We have a valid instrument for MR when a SNP affects proteins in a single path; in contrast, if an instrument is associated with proteins participating in different pathways, it violates the ‘exclusion restriction criterion’ and our instrument is invalid. (**B**) Integration of SNP–protein associations with pathway information and PPI data to distinguish vertical and horizontal pleiotropy using EpiGraphDB. All four proteins are associated with the same SNP. Proteins 1 and 2 share the same biological pathway. Proteins 2 and 3 are in PPI. Protein 4 shares no links with other proteins. Therefore, the SNP association on proteins 1, 2 and 3 are likely to act through vertical pleiotropy, where the SNP association on protein 4 verse other three proteins are likely to be horizontal pleiotropy

A key MR assumption is that the genetic variant [single-nucleotide polymorphism (SNP)] is only related to the outcome of interest through the exposure under study (the ‘exclusion restriction’ assumption). This assumption is potentially violated under horizontal pleiotropy, where a SNP is associated with multiple phenotypes (e.g. proteins) independently of the exposure of interest. In contrast, vertical pleiotropy, where a SNP is associated with multiple phenotypes on the causal pathway to the outcome, does not violate the ‘exclusion restriction criterion’ of MR (Fig. 2A). For molecular phenotypes, where the number of genetic instruments is typically limited, it is almost impossible to distinguish vertical and horizontal pleiotropy using established statistical approaches (van Kippersluis and Rietveld, 2018).

Here, by integrating SNP–protein associations with biological pathway and PPI information retrieved from EpiGraphDB, we have developed an approach to assess potential horizontal pleiotropy. As demonstrated in Figure 2B, for a SNP associated with a group of proteins, we check the number of biological pathways and PPIs that are shared across this group of proteins. If these proteins are mapped to the same biological pathway and/or a PPI exists between them, then the SNP is more likely to act through vertical pleiotropy and therefore be a valid instrument for MR.

#### 3.1.1Assessing the pleiotropy of an autoimmune-related variant

In this case study, we assessed the pleiotropy of rs12720356, a SNP located in TYK2 gene that is associated with Crohn’s disease and psoriasis (Solovieff *et al.*, 2013), by exploring the relationships between genes (and their products) to which this SNP can be mapped using expression QTL data. We used the GTEx database (The GTEx Consortium *et al.*, 2015) to identify single-tissue eQTL effects, gathering a set of genes whose expression level is associated with rs12720356 in different tissues: FDX1L, ICAM1, ICAM5, KRI1, MRPL4, GRAP2, TMED1, TYK2 and ZGLP (details on the list of pleiotropic genes are reported in Supplementary Table S4). We then proceeded to query EpiGraphDB to extract pathway and PPI data as described in the methods section (Supplementary Appendix S5).

**Fig. 3.**
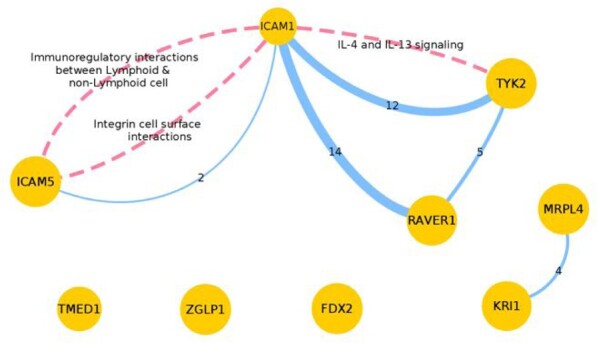
Network diagram with the evidence to assess the pleiotropy of genetic variant rs12720356. The network has one node for each protein regulated by the eQTL rs12720356, and their size is inversely proportional to their *P*-value (see Supplementary Table S4 for details). Dashed pink edges depict the participation in common biological pathways, and blue edges represent the number of shared PPIs (value indicated)

The results were then converted to a graph that shows two small connected components and a few isolated nodes (Fig. 3). Note that, the knowledge about biological processes described by pathways has to be considered incomplete, and perhaps partially incorrect, and therefore these results must be treated as hypothesis generating, and the user should be aware that absence of interaction evidence is not definitive proof of an absence of pleiotropy. Also, given that the same protein might participate in different pathways in different contexts, it is important to verify the soundness of these relationships. For instance, in our case study, ICAM1 shares pathways with ICAM5, the interactions of integrin cell surface and between lymphoid and non-lymphoid cells. Integrin expression has been shown to be altered in psoriasis (Creamer *et al.*, 1995), and integrins also have an important pro-inflammatory role in Crohn’s disease, where they facilitate the movement of leukocytes from the systemic circulation (note that the association is detected in whole blood) to the intestinal mucosa (Park and Jeen, 2018). ICAM1 also participates with TYK2 in the regulation of Interleukin-4 (IL4) and Interleukin-13 (IL3) signalling, important actors that drive a predominantly humorally mediated hypersensitivity response (Sartor, 1994). In terms of PPIs, the above pairs of genes are still connected, and we retrieved a triple formed by ICAM1, RAVER1 and TYK2, and the pair KRI1-MRPL4 that is associated with sun exposure, a well-established beneficial factor for psoriasis and Crohn’s disease (Jantchou *et al.*, 2014; Søyland *et al.*, 2011). However, here, the results depict that some single-tissue eQTLs with a strong association, like ZGLP1 and FDX2, remain unconnected in our network. This shows that they potentially work along different molecular pathways, acting in horizontal pleiotropy. It would be important to consider their potential biological role in the outcome phenotypes of any MR analyses using this instrument.

#### 3.1.2An exemplar valid instrument

We recently used the same approach to explore potential vertical and horizontal pleiotropy for a number of pleiotropic protein associated SNPs (Zheng *et al.*, 2020). In one example, a specific set of three proteins (IL6ST, ICAM1 and TIMP1) were associated with the same SNP (rs144276707). The pair ICAM1 and TIMP1 was found to participate in two common pathways, and there were four shared PPIs among all three proteins. These results supported the hypothesis that rs144276707 is more likely to influence these proteins via the same biological pathway (acting through vertical pleiotropy), strengthening the evidence that this SNP is a valid instrument for MR analysis.

### 3.2Identification of potential drug targets

Systematic MR of molecular phenotypes, such as proteins and levels of transcript expression, opens up important possibilities for drug–target prioritization in pharmacological investigations. However, many potential targets are not easily druggable. A parallel problem is that current GWAS of plasma proteins have limited sample sizes, are not available in many tissues, and only represent a subset of all proteins. A potential way to address these problems is to use PPI information to identify druggable targets linked to a non-druggable, but robustly causal gene. Their relationship to the causal gene increases our confidence in their potential causal role even if the initial evidence of their causal effect is below our multiple-testing threshold. Here, we have developed an approach using PPI data to prioritize potential alternative drug targets. As a proof of principle, we illustrate this approach using IL23R as an example.

#### 3.2.1Integrating MR evidence with PPI networks for alternative drug–targets search

IL23R is a well-established disease-susceptibility gene for inflammatory bowel disease (IBD) (de Lange *et al.*, 2017). The protein–disease association information retrieved from EpiGraphDB suggests that IL23R has a robust causal effect on IBD (https://epigraphdb.org/pqtl/IL23R) (beta = 1.50, *P*-value = 2.21 × 10^−166^, colocalization probability = 75%) (Zheng *et al.*, 2020). The drug PTG-200, acting as an antagonist of IL23R has just passed Phase I and is in Phase II trials for IBD treatment (Cheng *et al.*, 2019), which aligns well with the genetic/MR evidence implemented in EpiGraphDB. Whilst IL23R is druggable, we illustrate how our approach can identify potential alternative targets using pathway data.

We used PPI information (Orchard *et al.*, 2014; Szklarczyk *et al.*, 2019) and data on druggability (Finan *et al.*, 2017) to identify a set of proteins, which are the target of approved drugs and clinical-phase drug candidates and have direct PPI with IL23R. Table 2 shows a subset of this list with strong MR evidence (*P*-value <1 × 10^−5^) to IBD (Supplementary Table S5 reports the full list of identified proteins with druggability information).

**Table 2.** Triangulation of MR and literature evidence on the effects of IL23R and associated genes to IBD

Gene	Effect size (SE)	*P*-value	QTL	SemMed predicate (count)
IL23R	1.50 (0.05)	2.21 × 10^−166^	pQTL	AFFECTS (1), ASSOCIATED_WITH (21), NEG_ASSOCIATED_WITH (2), PREDISPOSES (1)
	0.89 (0.06)	4.16 × 10^−43^	eQTL	
IL12B	0.42 (0.03)	9.59 × 10^−34^	pQTL	ASSOCIATED_WITH (5)
IL15	−1.42 (0.20)	5.53 × 10^−13^	eQTL	ASSOCIATED_WITH (2)
IL4	0.46 (0.08)	4.47 × 10^−08^	eQTL	ASSOCIATED_WITH (3), DISRUPTS (1)
JAK2	−1.90 (0.20)	1.32 × 10^−20^	eQTL	AFFECTS (1), ASSOCIATED_WITH (3)
NFKB1	0.97 (0.17)	2.16 × 10^−08^	eQTL	ASSOCIATED_WITH (2)
RORC	−1.00 (0.12)	1.21 × 10^−17^	eQTL	ASSOCIATED_WITH (1)
STAT3	0.60 (0.08)	2.96 × 10^−15^	eQTL	AFFECTS (2), AUGMENTS (1), ASSOCIATED_WITH (9), CAUSES (1)

*Note*: The MR evidence is the QTL MR estimates of IL23R and the associated druggable genes (via direct PPI with Tier 1 druggability) to IBD GWAS (OpenGWAS ID: ieu-a-249). The literature evidence is the SemMed predicates derived by SemMedDB and the numbers of PubMed articles identified to support the predicate mechanism. Here, we report the subset of genes that are identified to contain both MR evidence (*P*-value <1 × 10^−5^).

This list of proteins includes IL12B, the target protein for an existing drug Ustekinumab, which is currently under Phase 3 and 4 trials for IBD treatment (drug trial information available via Open Targets https://www.targetvalidation.org/evidence/ENSG00000113302/EFO_0000540?view=sec:known_drug). Although there is strong MR evidence for IL12B (beta = 0.42, *P*-value = 9.59 × 10^−34^), there is little evidence for genetic colocalization (https://epigraphdb.org/pqtl/IL12B) (colocalization probability <1%), which prevents us prioritizing this target based on MR evidence alone. However, the PPI between IL12B and IL23R (which *does* have reliable MR and colocalization results) increases our confidence that IL12B is a valid target.

#### 3.2.2Using literature evidence for results enrichment and triangulation

A further source of useful evidence is the literature-derived knowledge from SemMedDB (Kilicoglu *et al.*, 2012) available in EpiGraphDB. Integrating this literature evidence with the evidence described above can further enhance confidence in the findings (as well as identify potential alternative drug targets). Table 2 also reports the gene-to-trait literature evidence regarding IL23R and interacting proteins and IBD, where each entry shows a literature-derived semantic triple (e.g. ‘IL23R’—‘ASSOCIATED_WITH’—‘Inflammatory Bowel Diseases’), as well as the study articles from which each triple was extracted. For the list of genes including IL23R and IL12B that were identified with strong MR evidence, we were also able to find abundant literature evidence supporting the genetic causal evidence with derived mechanisms involving predicates, such as ASSOCIATED_WITH, AFFECTS and CAUSES.

### 3.3Triangulating causal estimates with literature evidence

Previously, we have demonstrated that existing literature can be used to derive relationships and mechanisms between defined biomedical traits (Elsworth *et al.*, 2018; Elsworth and Gaunt, 2020). By integrating this knowledge with causal estimates in EpiGraphDB, we can triangulate evidence, identifying where these two sources of evidence are in agreement, and where they are not (Lawlor *et al.*, 2017). In this case study, we explore the literature connecting traits with pre-defined causal relationships. From here, we can summarize the key mechanisms defined in the literature, and also potentially derive novel mechanisms.

#### 3.3.1Sleep duration and coronary heart disease as an example

Starting with an exposure trait of ‘Sleep duration’, existing MR data, and connections between traits and diseases in EpiGraphDB, we extracted a set of potentially causally related traits (note that this is limited to traits with GWAS and MR results) (Table 3).Multiple disease entries arise from the mapping between the trait name and EFO terms, each of which maps to a disease term. In this case, we treated each mapping as a single relationship and extracted the literature data connecting a pair of traits. For this example, we selected the outcome trait ‘Coronary heart disease’ to explore in more detail the potential mechanisms linking this to sleep duration. To do this, we queried EpiGraphDB to extract the semantic triples associated with each trait and searched for overlapping terms, identifying 839 overlapping triples (Supplementary Table S6 reports the top 10 items by enrichment *P*-value).

**Table 3.** Summary of disease traits identified with causal association to ‘Sleep duration’

Exposure	Outcome	MR beta	MR *P*-value	Disease
ieu-a-1088: sleep duration	ukb-a-107: non-cancer illness code self-reported: gout	−0.00257	3.8 × 10^−24^	‘gout’
ieu-a-1088: sleep duration	ieu-a-6: coronary heart disease	−1.03933	2.3 × 10^−21^	‘coronary artery disease’
ieu-a-1088: sleep duration	ukb-a-548: Diagnoses - main ICD10: K35 acute appendicitis	−0.00671	8.0 × 10^−15^	‘appendicitis’
ieu-a-1088: sleep duration	ukb-a-54: cancer code self-reported: lung cancer	−0.00191	1.1 × 10^−14^	‘cancer’, ‘lung carcinoma’
ukb-a-9: sleep duration	ukb-a-13: sleeplessness/insomnia	−0.32167	1.1 × 10^−11^	‘insomnia (disease)’

*Note*: We searched for MR evidence associated with the trait ‘Sleep duration’ with *P*-value to be under 1 × 10^−10^, and map the outcome trait to a disease term via mappings through EFO terms. The identifiers ‘*ieu-a-*’*/*‘*ukb-a-*’ are IEU OpenGWAS IDs.

**Fig. 4.**
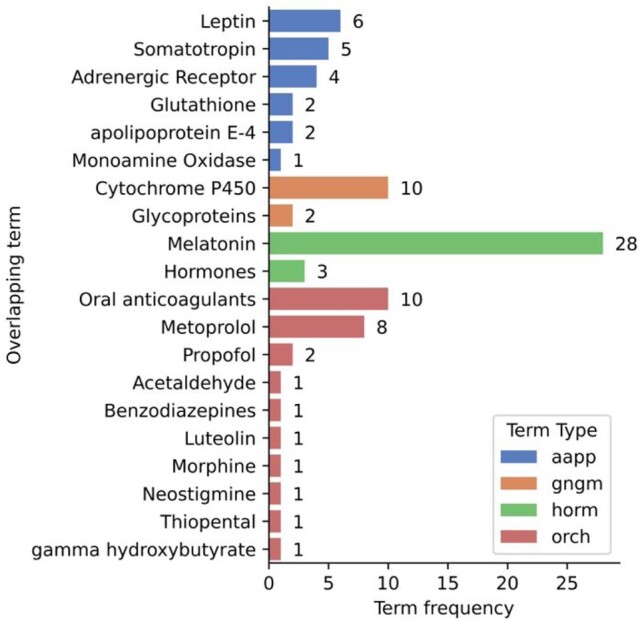
Literature-mined/derived evidence on the intermediates between ‘Sleep duration’ and ‘Coronary heart disease’. Counts of overlapping SemMed terms grouped by the SemMed term type (aapp—amino acids, peptides, proteins, gngm—genes or genome, horm—hormones, orch—organic chemicals; full list available at https://mmtx.nlm.nih.gov/MMTx/semanticTypes.shtml)

We then generated frequency counts for the overlapping terms (Fig. 4), which identified many different overlapping terms and types (https://mmtx.nlm.nih.gov/MMTx/semanticTypes.shtml), including 6 proteins (*aapp*), 2 genes (*gngm)* and 11 organic chemicals (*orch*). Each of these represents a key point in a potential mechanism, connecting the exposure and outcome traits. Terms of particular interest are those with high counts (e.g. Ethanol) as these represent terms with large numbers of supporting publications in the literature. However, in this case, Ethanol may be present in such numbers due to its inclusion in many publications as a co-factor when describing the functionality and efficacy of drugs, highlighting the importance of reviewing a selection of articles underpinning each mechanism. For this reason, Ethanol has been excluded from this example.

**Fig. 5.**
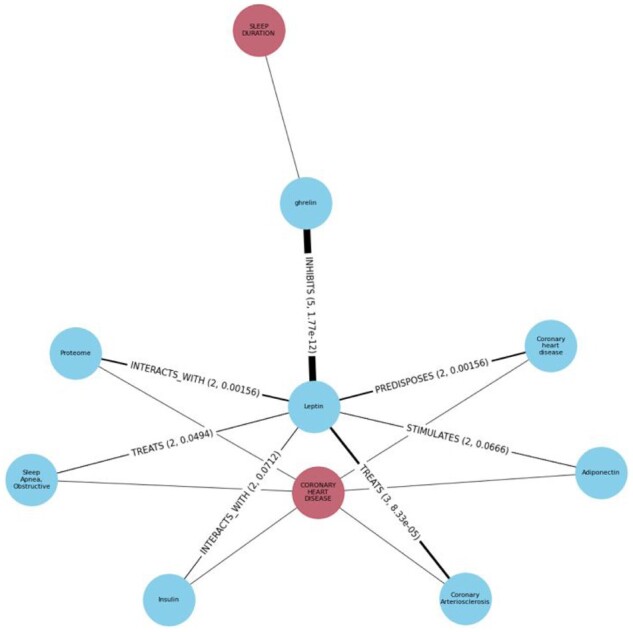
Literature-derived mechanisms between ‘Sleep duration’, ‘Leptin’ and ‘Coronary Heart Disease’. Network diagram displaying the literature connections between ‘Sleep Duration’ and ‘Coronary Heart Disease’ through the intermediate term ‘Leptin’. Predicates connecting two semantic terms, their frequencies and enrichment *P*-value are labelled on the edges. Enrichment is calculated via MELODI Presto (Elsworth and Gaunt, 2020) based on a comparison of query count to background. Edge width represents the enrichment log transformed *P*-value. Red nodes represent the exposure (SLEEP DURATION) and outcome (CORONARY HEART DISEASE) traits, blue nodes represent intermediate semantic literature nodes

Figure 5 suggests the main route from Sleep Duration to Coronary Heart Disease via the intermediate term Leptin involves only one term on the exposure side (‘ghrelin’) and 10 on the outcome side, the most enriched being ‘Leptin – TREATS - Coronary Arteriosclerosis’, providing this as a potential mechanism of interest.

#### 3.3.2Check findings against the original publication text

Finally, EpiGraphDB provides a PubMed identifier to enable us to check the validity of these connections in the original text. For example, we found evidence of two statements that ‘Leptin PREDISPOSES Coronary heart disease’. These were derived from the following two sentences:

‘CONCLUSIONS: Consumption of sugar-sweetened beverages was associated with increased risk of CHD and some adverse changes in lipids, inflammatory factors, and leptin’ (de Koning *et al.*, 2012).

‘Leptin, one of the earlier adipocytokines, is known to play a major role in cardiovascular disease and recent observations suggest that leptin is an independent risk factor for coronary heart disease’ (Amasyali *et al.*, 2010).

The contrasting causal interpretation of these two sentences highlights the importance of manual review of the original articles to validate the semantic triples.

## 4 Discussion

EpiGraphDB is a new database and platform for data integration in health data science, with a particular focus on understanding the relationships between risk factors, intermediate phenotypes and disease outcomes described by epidemiological analyses. Whilst, we present three specific case studies, we anticipate a much wider array of uses and support this through an open API and R package. It is, however, important to recognize that there are several existing platforms for data integration in the health, biomedical and pharmaceutical domains (Supplementary Table S3).

The Open Targets platform (Carvalho-Silva *et al.*, 2019; Koscielny *et al.*, 2017) (https://www.targetvalidation.org/) integrates a wealth of genomic, phenotypic, ontology and drug–target data into a single platform aimed at users in the pharmaceutical industry and research community. Their platform has a well-developed web interface in addition to a comprehensive API and Python package to support the use of the API. This open approach has enabled EpiGraphDB to utilize drug/target mappings with Open Targets. However, whilst there is some overlap in this context, the Open Targets platform lacks MR estimates (although it does include genetic association data). Open Targets also includes some literature data, and their LINK platform (https://link.opentargets.io/) extracts semantic relationships from PubMed. However, despite some of the conceptual similarities to EpiGraphDB, their focus is primarily on drug–target prioritization, whilst EpiGraphDB also aims to support the evaluation of lifestyle risk factors.

The Hetionet platform (https://het.io/) is a graph database integrating data from more than 29 different databases, which was initially set up to prioritize drugs for repurposing using an innovative approach to predict gene/disease associations (‘Project Rephetio’) (Himmelstein *et al.*, 2017; Himmelstein and Baranzini, 2015), but now aims to have a broader remit. The platform is very accessible, with a web application, data downloads in multiple formats and open access to their Neo4j database. The primary focus of the platform is for molecular mechanisms and pharmacologic data while EpiGraphDB additionally encompasses epidemiological relationships (MR causal estimates, genetic correlation, etc.) and literature data. However, the open nature of the platform enables users to easily work with Hetionet in parallel with EpiGraphDB.

The Monarch Initiative (Mungall *et al.*, 2017) (https://monarchinitiative.org/) is focussed on the integration of genotypic and phenotypic data across species with the aim of identifying related phenotypes and potential animal models of disease. This contrasts with the human-centric epidemiological focus of EpiGraphDB. The Monarch Initiative platform has an open-source approach to software development and offers web interfaces powered by an open API. In common with Hetionet and EpiGraphDB, the platform uses the Neo4j database. Users can easily integrate data from the Monarch Initiative with EpiGraphDB given their open design principles.

Wikidata (https://wikidata.org) is a general knowledge base which contains an array of biomedical data sources that have recently been reported (Waagmeester *et al.*, 2020). In contrast to curated knowledge bases, such as EpiGraphDB, Wikidata is developed through community-driven efforts and bot automation, and incorporates extensive knowledge across a wide array of fields, including (but not limited to) a range of biomedical entities. Unfortunately, the scope of this project leads to inevitable duplication and redundancy of the entities it comprises. This much broader approach distinguishes Wikidata from specialist platforms, such as EpiGraphDB, which is focussed on epidemiological and biomedical knowledge. In common with other platforms listed above, the open design of this platform supports cross-platform data integration.

Various other platforms (Abbot *et al.*, 2020; Coker *et al.*, 2019; Gaspar *et al.*, 2018) exist with some conceptual overlaps with EpiGraphDB (Supplementary Table S3). These represent a range of different types of data based on molecular and genetic interactions and drug targets. However, in contrast to the platforms described above these platforms do not appear to have accessible API or software packages. Although several are open access and available to the wider community, the lack of programmatic interoperability limits their scope.

As with all similar platforms, EpiGraphDB is constrained by the available data and subject to any errors or quality issues that exist in the original sources. However, by integrating data from a range of sources (e.g. STRING, IntAct and Reactome for interactions between proteins), we ensure the user can evaluate consistency between data sources. We welcome feedback and suggestions from users (https://docs.epigraphdb.org/#contact) and we provide information on upcoming additions and updates via the platform documentation (https://docs.epigraphdb.org/CHANGELOG/).

## 5 Conclusions

The EpiGraphDB platform provides an integrated data resource to support data mining and interpretation of the relationships between disease risk factors, intervention targets and disease outcomes. We present three illustrative case studies that demonstrate the functionality and utility of the platform, but it is important to note that much more extensive capabilities are available and will continue to expand as the platform is developed further. We aim to support open science by making the data freely accessible, both programmatically and through a web interface, and by providing open-source code and exemplar Jupyter notebooks.

## Author contribution

Y.L., B.E., P.E., V.H., O.L., M.V. and T.R.G. wrote and edited the manuscript. Y.L., B.E. and T.R.G. developed the platform architecture, and Y.L., B.E., T.R.G., V.H., O.L. and M.V. developed the platform components. B.E. developed the data integration pipeline, and B.E., Y.L., P.E., V.H., G.H., M.L. and J.Z. were responsible for the data generation, acquisition and curation. Y.L., P.E., V.H., B.E. and J.Z. developed the case studies. T.R.G. conceptualized and supervised the project. All authors contributed to the review of the article and provided comments.

## Funding

This work was supported by the UK Medical Research Council [MC_UU_00011/4]. J.Z. is a University of Bristol Vice-Chancellors Fellow. G.H. was funded by the Wellcome Trust and Royal Society [208806/Z/17/Z]. This work has also been supported by a Cancer Research UK programme grant [C18281/A19169] and British Heart Foundation Accelerator Award [AA/18/7/34219]. This work has also been supported by the NIHR Biomedical Research Centre at University Hospitals Bristol and Weston NHS Foundation Trust and the University of Bristol. T.R.G. and G.H. receive research funding from GlaxoSmithKline and Biogen. V.H. has previously been supported by funding from GlaxoSmithKline.

*Conflict of Interest*: none declared.

## Supplementary Material

btaa961_Supplementary_Data
