# Erratum to: EpiGraphDB: a database and data mining platform for health data science

**DOI:** 10.1093/bioinformatics/btab104

**Published:** 2021-03-10

**Authors:** Yi Liu, Benjamin Elsworth, Pau Erola, Valeriia Haberland, Gibran Hemani, Matt Lyon, Jie Zheng, Oliver Lloyd, Marina Vabistsevits, Tom R Gaunt

*Bioinformatics* (2020) doi: 10.1093/bioinformatics/btaa961

Upon the original publication of this article, there was an error in the source code syntax under sub-section “2.2 Integration of epidemiological evidence” in the “Materials and methods” section. The source code syntax should read: “(e.g. (Gwas {trait: ‘Body mass index’})-[MR {beta, se, pval}]->(Gwas {trait: ‘Coronary heart disease’}))” instead of “ (e.g. [Gwas (trait: ‘Body mass index’)]-[MR {beta, se, pval}]->(Gwas {trait: ‘Coronary heart disease’})))”. This error has now been corrected. The Publisher apologizes for the error.

